# Investigating Father or Partner Involvement in Family Integrated Care in Neonatal Units: Protocol for a Prospective, Multicenter, Multiphase Study

**DOI:** 10.2196/53160

**Published:** 2024-03-25

**Authors:** Rupa Rubinstein, Katie Gallagher, John Ho, Julian Bose, Minesh Khashu, Narendra Aladangady

**Affiliations:** 1 Neonatal Unit Homerton Healthcare NHS Foundation Trust London United Kingdom; 2 Blizard Institute Queen Mary University London London United Kingdom; 3 Institute of Women's Health University College London London United Kingdom; 4 Neonatal Unit Whipps Cross University Hospital Barts Health London United Kingdom; 5 Inspire Cornwall Community Interest Company's DadPad The Health and Wellbring Innovation Centre Truro United Kingdom; 6 Neonatal Unit University Hospitals Dorset NHS Foundation Trust Dorset United Kingdom

**Keywords:** family integrated care, FICare, neonatal intensive care unit, NICU, fathers, premature infants, mental health, pediatric, pediatrics, infant, infants, infancy, baby, babies, neonate, neonates, neonatal, premature, partner, couple, family care, NU admission, NICU admission, engagement

## Abstract

**Background:**

Neonatal unit (NU) admissions for premature babies can last for months, which can significantly impact parental mental health (MH) with symptoms of depression, stress, and anxiety. Literature suggests fathers experience comparable MH symptoms to mothers. Family integrated care (FICare) is a culture where parents are collaborators and partners in caring for their hospitalized newborns. FICare improves infant outcomes and maternal MH. Similar reports on fathers are limited.

**Objective:**

The primary aim of this study is to investigate the impact of supporting father or partner engagement in FICare of preterm infants on their MH up to 6 weeks postdischarge. The secondary aim is to investigate the impact on maternal MH.

**Methods:**

This is a 2-phase study: phase 1 to gather baseline information and phase 2 to assess the impact of enhanced father or partner engagement in FICare on their MH, involving 2 NUs (tertiary and level 2). Enhanced FICare will be developed and introduced (eg, information booklet, workbook, classes, and a father peer-support group) alongside standard FICare practices. Father or partner MH will be assessed with semistructured qualitative interviews and validated questionnaires: Generalized Anxiety Disorder Assessment, Patient Health Questionnaire, and Parental Stressor Scale: Neonatal Intensive Care Unit from NU admission to 6 weeks postdischarge. Mothers will be assessed by focus groups and the same questionnaires. Descriptive statistics and appropriate comparative tests, such as the 2-tailed *t* test, will be used to analyze and compare phase 1 and 2 data. Qualitative data will be coded line by line with the use of NVivo (Lumivero) and thematically analyzed. Simultaneously, systematic reviews (SRs) of fathers’ experiences of FICare and their MH outcomes will be conducted. The study was approved by the National Research Ethics Committee (22/EM/0140) in August 2022. A parent advisory group was formed to advise on the study methodology, materials, involvement of participant parents, and dissemination of study findings.

**Results:**

A recent SR demonstrated that data saturation is likely to be achieved by interviewing 9 to 17 participants. We will study a maximum of 20 parents of infants born at less than 33 weeks’ gestation in each phase. As of October 2023, the study was ongoing. The SR studies are registered with the PROSPERO database (324275 and 306760). The projected end date for data collection is July 2024; data analysis will be conducted in November 2024 and publication will occur in 2025.

**Conclusions:**

The study aims to demonstrate the feasibility of using a father or partner-sensitive FICare model for parents of premature babies with a positive impact on their MH. It will demonstrate the feasibility of providing FICare to extremely premature babies receiving intensive care. This study may support the development of inclusive FICare guidelines for nonbirthing parents and their extremely premature infants.

**Trial Registration:**

ClinicalTrials.gov: NCT06022991; https://classic.clinicaltrials.gov/ct2/show/NCT06022991

**International Registered Report Identifier (IRRID):**

DERR1-10.2196/53160

## Introduction

In 2019, in England and Wales, 49,489 premature births were recorded, and the majority would have required a neonatal unit (NU) admission [1]. Scientific and technological advancements have led to significant reductions in mortality and morbidity for preterm infants [2], and now the importance of decreasing psychosocial morbidity for the family unit is being addressed. Admission of a critically ill baby to the NU is a deeply traumatic time for parents and may result in parental mental health (MH) difficulties during and after discharge [3,4]. Studies exploring paternal experiences in the NU highlight fathers feeling neglected and in a unique position of stress, trying to balance work and home life to allow the mother to focus on their sick baby [5]. As paternal and maternal MH are closely linked, supporting the fathers could also positively impact maternal MH [6,7]. A recent study has shown reduced paternal stress in relation to the provision of family integrated care (FICare) in single-family rooms, but it only included parents of babies who did not require intensive care [8].

FICare in the NU means that parents are partners and collaborators in the care of their hospitalized baby, rather than observers or visitors. They are included in decision-making and supported to feed, clean, and hold their baby as well as learning how to provide neurodevelopmental care [9,10]. FICare studies that have focused on mothers and infants demonstrate improved neurodevelopmental outcomes [11], improved breastfeeding rates [12], reduced infection rates, and improved maternal MH [13,14]. There is a paucity of similar research regarding fathers [15]. The impact of parental MH on children is a focus of the UK government’s “First 1000 days of life” paper [16]. Implementation and improvements to FICare as a method of enhancing “parenting opportunity” are also highlighted in the National Neonatal Critical Care Review in England [17]. Synthesizing the evidence base surrounding the impact of paternal engagement addresses recommendations from National Health Service (NHS) England, the Cross Government 1001 Critical Days Campaign [7], the Neonatal Critical Care Transformation Review, and Care Quality Commission standards to increase the evidence base to support women and their families experiencing perinatal MH problems. This area currently costs the NHS an estimated £1.2 billion (equivalent to US $1.5 billion) per year [16,17]. The UK government has recently recognized the importance of maternal and paternal presence for babies in the NU via the Neonatal Care (Leave and Pay) Act, which achieved royal assent in May 2023 granting parents up to an additional 12 weeks of leave [18]. Hence, we developed a study protocol to investigate and enhance fathers’ or partners’ involvement in FICare and its impact on their MH.

This study will be the first to report on fathers’ experiences of FICare in the NU with extremely premature babies. It will broaden the field of FICare research with respect to fathers from diverse social and cultural backgrounds and will highlight the needs of fathers in the NU. Therefore, it will demonstrate the value of designing a father- or partner-sensitive FICare program. This study will also generate evidence for the feasibility of the introduction of FICare from day 1 while preterm babies are receiving intensive care.

From here on, although the study refers to “fathers,” all second parents of any gender or relationship status are included under this term throughout the remaining text.

## Methods

### Aims

This study aims to undertake an in-depth longitudinal study of the engagement of fathers or partners in FiCare and their experience and MH from NU admission to 6 weeks postdischarge of their premature baby born at 22 to 33 weeks’ gestation.

### Objectives

Our objectives are to (1) assess baseline fathers’ experiences in the NU, their involvement in FICare, and MH status during babies NU admission up to 6 weeks postdischarge, (2) conduct systematic reviews (SRs) of published papers on “Fathers and FiCare in NU” and “Fathers’ MH in the NU,” (3) develop a program to enhance the involvement of fathers in FiCare, and (4) introduce the program developed to enhance fathers’ involvement in FiCare and explore their experiences and effects on their MH.

### Outcomes

The primary outcome is paternal MH (stress) assessed by semistructured interviews.

The secondary outcomes are (1) maternal stress, assessed by the Parental Stressor Scale: Neonatal Intensive Care Unit (PSS:NICU) questionnaire; (2) paternal stress, assessed by the PSS:NICU questionnaire; (3) parental anxiety, assessed by the Generalized Anxiety Disorder Assessment-7 (GAD-7); (4) parental depression, assessed by the Personal Health Questionnaire-9 (PHQ-9); and (5); paternal engagement with FICare, assessed via interviews.

### Study Design

**Figure 1:**
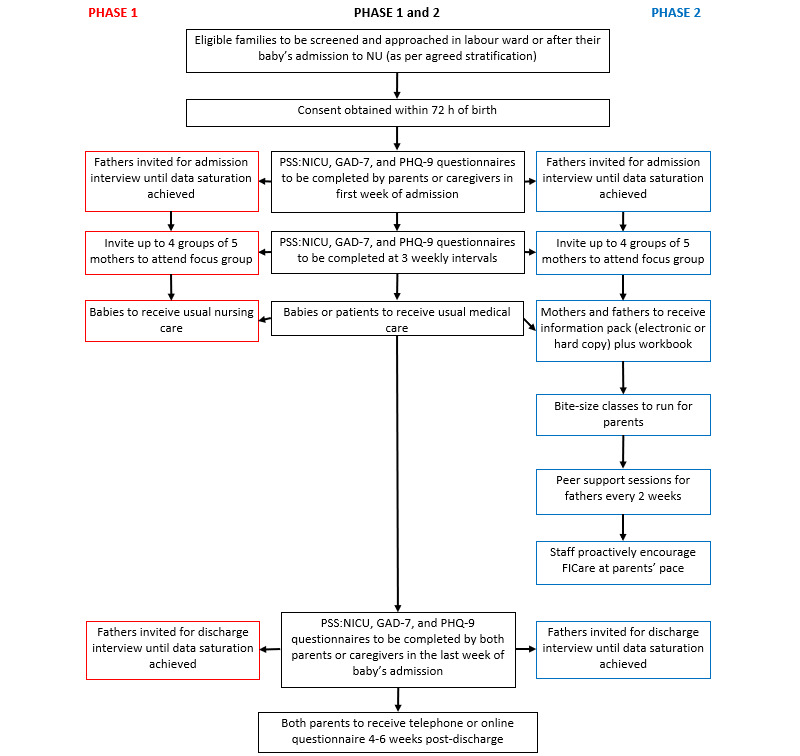
Participant flow chart. FICare: family integrated care; GAD-7: Generalized Anxiety Disorder-7; NU: neonatal unit; PHQ-9: Patient Health Questionnaire-9; PSS:NICU: Parental Stressor Scale: Neonatal Intensive Care Unit.

This is a prospective, multicenter, and multiphase study. Phase 1 will assess baseline fathers’ experiences in the NU, their involvement in FICare, and MH status during babies’ NU admission up to 6 weeks postdischarge. Simultaneously, an SR of fathers’ involvement in FICare and their MH outcomes will be conducted. A program to enhance the involvement of fathers in FICare will be developed using information from the phase 1 study, SR, existing DadPad or Imperial Family Delivered Neonatal Care app, and advice from the parent advisory group (PAG). In the phase 2 study, an enhanced father involvement program in FICare will be implemented and similar assessments as in phase 1 will be conducted (Figure 1). We will provide regular debrief sessions, and research updates or newsletters to keep staff engaged and supportive of the project. This support will be continued throughout the study.

This study has been registered with clinicaltrials.gov (NCT06022991) [19].

### Systematic Reviews

The SR studies have been registered on the PROSPERO database (324275 [20], 306760 [21]) and will follow a similar methodology. A literature search of health care databases including EMBASE, EMCARE, CINAHL, Medline, BNI, PYCHInfo, and Psychology and Behaviour, and a gray literature search will be conducted. Two independent researchers will screen the results at the title and abstract level against the eligibility criteria (Table 1), with mediation by a third researcher available for any disagreements to reevaluate the paper and come to a team agreement. The matrix method will be used for data extraction [22].

**Table 1:** Eligibility criteria for the systematic reviews.

	Fathers’ mental health in the NU^a^ [18]	Fathers and FICare^b^ in the NU [19]
Studies published from January 1, 2000, to October 14, 2022	✓	✓
Full paper available in English	✓	✓
Study setting is the NU	✓	✓
At least 5 study participants	✓	✓
At least half of the participants are fathers	✓	✓
Addressed paternal mental health	✓	
Addressed FICare		✓

^a^NU: neonatal unit.

^b^FICare: family integrated care.

### Study Centers

The study will be conducted at a tertiary NU (level 3) and a local NU (level 2), as defined by the Department of Health [23], in East London, United Kingdom. Average annual admissions are approximately 900 and 600 respectively, with parents from diverse ethnic and socioeconomic backgrounds. Both level 3 and 2 NUs are selected to show the feasibility of delivering enhanced father involvement in FICare in all NUs and the study findings are applicable to all levels of NUs. Although FICare culture is practiced at both sites, a formal program and expectations of FICare are not implemented.

### Clinical Staff Involvement

The wider multidisciplinary team was consulted in the study design and consideration of phase 2 study materials. They included physiotherapists, clinical psychologists, occupational therapists, dieticians, and speech and language therapists along with neonatal nursing input. The London neonatal network care coordinator was also consulted regarding the study design and implementation.

### Patient and Public Involvement

Patients and the public were involved in the design, conduct, reporting, and dissemination plans of the study. A PAG consisting of parents of preterm infants was developed. They consisted of a diverse socioeconomic and cultural mix of 4 fathers and a mother who had experienced the admission of their premature baby in the NU within the last 4 years. The PAG was involved in developing the study design. The PAG has reviewed and provided feedback on study materials, for example, participant information leaflet. A lay member is included as a coinvestigator (JB, Director of Inspire Cornwall CIC’s Neonatal DadPad) of the study.

The PAG was consulted to advise on the burden of intervention and the time required to participate in the research. This has influenced the frequency of questionnaire administration and locations of interviews and focus groups. They will support the development of appropriate phase 2 materials for the information booklet, workbook, and education sessions. The PAG will also support the dissemination of the study findings to research participants, related charity bodies (eg, Bliss), and the wider neonatal community. 

### Study Participants and Recruitment

Parents of preterm infants will be recruited to the study.

#### Inclusion Criteria

The inclusion criteria are as follows: (1) parents of 22^+0^ to 32^+6^ weeks preterm infants; (2) infants aged 1 to 7 days; (3) infants with 2 primary caregivers (the father’s or partner’s participation in the study is a must, and participation from both parents is ideal), and (4) the parents should have conversational English.

#### Exclusion Criteria

The inclusion criteria are as follows: (1) infant with life-limiting conditions or no realistic chance of survival, (2) infant is likely to be transferred to nonparticipating center within 4 weeks of age, and (3) parents are younger than 16 years.

All parents with babies born between 22^+0^ and 32^+6^ weeks’ gestation admitted to the participating centers at less than 7 days of age will be assessed for eligibility. A sequential recruitment strategy will be followed with all eligible parents from the date of recruitment opening until 20 parents have been recruited in each phase. Within each eligible parent, consent and recruitment of the father or partner is essential. Maternal recruitment in addition to the partner, is encouraged but not mandatory.

#### Sample Size

The sample size of the study is estimated based on qualitative study outcomes. A recent SR of qualitative study demonstrated that to achieve data saturation we need to interview 9 to 17 participants [24]. A focus group of 5 to 7 participants would be enough for in-depth conversations and 2 or more groups would increase the chances of successful data output [25].

In the first and second phases, up to 20 parents will be recruited (until the fathers’ interviews reach data saturation). With a father and mother each, there could be 40 participants per phase. In total, there could be 80 participants in the study.

### Phase 1 Study: Baseline Data Collection (9 Months)

#### Overview

Phase 1 will be conducted as follows: (1) Prior to the introduction of the study to wider NU clinical staff members, staff knowledge, experiences, and opinions about the role of fathers and FICare will be assessed by a questionnaire. (2) Once a parent is recruited, the PSS:NICU [26] and MH questionnaires (GAD-7 [27] and PHQ-9 [28]) will be given to complete within 7 days. (3) Fathers or partners will be invited to participate in 2 semistructured interviews, within 2 weeks of recruitment and immediately prior to discharge. The interviews will explore their MH, experiences of bonding with baby, understanding the neonatal environment, and reflecting on their service expectations. Based on the father’s or partner’s preference, interviews will be undertaken either in a private room on the NU or digitally via MS Teams at a mutually convenient time. Interviews will be digitally recorded and are anticipated to last up to 1 hour. Data saturation is expected at around 12-15 fathers. (4) The parents will be asked to complete the PSS:NICU, GAD-7, and PHQ-9 questionnaires at 3 weekly intervals, finishing on the week of discharge, to monitor their MH through their NU stay. (5) Up to 4 groups of 5 mothers will be invited to participate in a focus group to assess their wellbeing, experience of the NU, and their perspective on support for and from their partners. This will occur in a room in the NU at a time convenient to the mothers. Groups will be organized until data saturation is reached. (6) Telephone interviews and emailed questionnaires will be completed by parents at 4-6 weeks postdischarge to assess family confidence and bonding after NU discharge.

#### Description of Questionnaires Used

##### PSS:NICU Questionnaire

The PSS:NICU was originally developed in 1993 [26]. It assesses neonatal intensive care unit–specific stressors on a Likert scale. Each item can be scored from 0 to 5, with a maximum total score of 230 over the 46 items. Modified versions exist, but the original full-length questionnaire will be used for this study as all subsections are of interest with changes to be implemented. 

##### GAD-7 Questionnaire

The GAD-7 is a 7-item self-administered questionnaire that assesses the presence and severity of symptoms of anxiety over the preceding fortnight. Scores range from 0 to 3 on each item, with a total score ranging from 0 to 21. There are established set points for mild, moderate, and severe anxiety [27].

##### PHQ-9 Questionnaire

The PHQ-9 is a 9-item self-administered questionnaire that assesses the presence and severity of symptoms of depression over the previous two weeks. It is scored in the same manner as the GAD-7 with total scores ranging from 0 to 27 and with established set points for mild, moderate, and severe symptoms [28].

### Washout Period, Phase 2 Preparation (4 Months)

A program to enhance father participation with the FICare model will be developed using the existing Neonatal DadPad or Imperial Family Delivered Neonatal Care app. Both of these apps are currently used in several NHS Trusts to guide fathers through their NU journey. The phase 1 and systematic review findings, along with guidance from the PAG will inform our decision for what is best suited for our local population.

Teaching sessions will be undertaken to educate and upskill all clinical NU staff on the study aims, recruitment, phase 1 study findings, and upcoming phase 2 study, highlighting the importance of fathers’ role in the FICare (eg, teaching parents how to safely give nasogastric feeds and nappy changes in a ventilated baby). In addition, there will be regular FICare teaching for staff, including encouragement of parents to attend ward rounds, and with guidance to parents in the workbook (Table 2).

**Table 2:** Program to enhance father involvement in family integrated care.

Strategies	When	Who
Educational booklet or app, including Introduction to the neonatal unit Meeting the multidisciplinary team—tailored to the individual unit Family integrated care Taking care of yourselves Routine care and “observations” Breastfeeding Developmental care Medications Nutrition and growth Ventilation and breathing support Cardiac (heart) conditions Gastrointestinal and liver (gut) conditions Brain development Planning for discharge Eyes	At parents’ disposal	Both parents
Suggested tasks or workbook, including Kangaroo care Nappy changes in incubator Mouth care and cleaning of baby Reading of observations (heart rate, temperature, respiratory rate) Helping to weigh baby Bathing baby Ward round participation	To work through at parents’ pace	Both parents
Peer support sessions—led by specialist clinical psychologists or therapists	Every two weeks	Fathers^a,b^
Ward round participation	Daily	Both parents^a^
Bite-size education classes	2-3 per week at varying times including weekend	Both parents^a,b^

^a^Any parent in the unit is welcome to participate or attend.

^b^Sessions to occur at times to suit fathers with cultural, religious, family, and work obligations, as determined through phase 1 data collection.

### Phase 2 Study: Enhanced Father Involvement in FICare (9 Months)

Once a family is recruited, the program developed to enhance the father’s involvement in FICare will be implemented to take place at the parents’ pace (Table 2). Materials will be freely available to the study participants in a format suited to their needs (electronic or paper copy). All interested parents are encouraged to attend the peer support sessions, ward round participation, and bite-size education classes.

The NU experience and MH status of both parents will be assessed as in the phase 1 study (Figure 1) including assessing their experience of the FICare program. At the end of the phase 2 study, staff knowledge, experiences, and opinions about fathers and FICare will be reassessed and compared with the start of the study.

### Statistical Analysis

Qualitative data will be imported into dedicated software packages to facilitate analysis including NVivo (Lumivere) and SPSS (SPSS Inc). Interviews will be transcribed, following which the original recordings will be destroyed.

### Interviews and Focus Groups

Data will be uploaded into NVivo to facilitate qualitative data management and thematic analysis. We will use several methods to minimize researcher bias during the study. All interviews will be conducted by 1 person (RR), who will maintain a reflexive diary through the study process. The transcripts will be independently coded by 2 researchers (RR and KG) to find repeated patterns of meaning which provide a detailed understanding of the perspective of the participants [29]. Both researchers will systematically code the data in detail and group them together into broader themes, with themes revisited to ensure that data saturation has been reached. These will then be grouped into broader themes generating a thematic map that will allow us to understand the parents’ experiences in NU. The chief investigator (NA) will have control of the data and will be able to access the audio recordings of the interviews at all times to audit the quality of the interview process. Decision trails and coding trees will be used to ensure that coding and decision-making in data analysis are clear and transparent. Any discrepancies will be discussed with a third person (NA). Verbatim (anonymous) quotes will be shared with the results to allow the reader to determine whether the final themes reflect the data. Finally, the research team and participants will be invited to comment on the emerging or final themes during the data analysis.

### Questionnaires

For descriptive statistics, the median and mode scores of the PSS:NICU, GAD-7, and PHQ-9 will be used to describe the outcome of the questionnaires. For inference analysis, the difference in median for the PSS:NICU, GAD-7, and PHQ-9 at admission, discharge, and 6 weeks postdischarge will be compared between phase 1 and phase 2 using the Mann-Whitney test and the paired 2-tailed *t* test. The data will be checked for outliers and these will be corrected or removed. However, where these outliers are accurate, a nonparametric statistical analysis will be performed. Depending on the data type, missing data will be replaced with mean, median, or mode. In addition, regression and multiple imputations analysis will also be used to impute or generate missing values.

Staff survey questionnaire responses to individual questions will be presented as a percentage of individuals responding yes or no. The percentage of “yes” responses to each question by the staff at the start and end of the study will be compared by paired *t* test.

### Data Storage and Availability

All patient-identifiable data will be stored in a locked office of the principal investigator (PI) of participating sites. Access to identifiable data will be restricted to the PI and research team. The paper questionnaires will be stored securely in a file kept in the locked research office and electronic versions will be stored in a password-protected NHS computer. The electronic version of questionnaires will be emailed by parents to a secure NHS email account. This will also be used if transcription or audio files need to be transferred with third-party transcription services. Third-party services will be vetted as per NHS standards to ensure data protection and confidentiality. The sponsor will have access to the pseudoanonymized data if requested. Anonymized data will be made available on request. Study data will be kept for up to 18 months after the study completion.

### Potential Risk to Participants and Mitigation

Considering the nature of the study, it is unlikely to have any adverse events. However, fathers may feel the additional burden of fulfilling their expected NU obligations alongside their breadwinner role and feelings of guilt for being unable to fulfill the expectations set for them [30,31]. To mitigate this difficulty, there is flexibility in when and to what extent fathers would like to be involved in FICare set at their own pace. The questionnaires and interviews may be emotionally challenging. This is being mitigated by the option to attend peer support sessions run by an appropriately trained professional and all parents will have access to the local neonatal psychologist. Interview frameworks were reviewed by the PAG and neonatal psychologist. A protocol is in place for any participants exhibiting severe MH symptoms through questionnaire responses or other forms of communication. Adverse events will be reported to the chief investigator and local research and development office and follow research and development, protocols with consideration of modification of study procedures if required.

Adverse events in the hospital setting will follow local hospital protocols as well as escalation to study PI with consideration of modification of study procedures if required. The risk of psychological distress resulting from study participation has been minimized with the support of a specialist psychotherapist who reviewed the study design and materials. A protocol is in place for any parents exhibiting severe MH symptoms through questionnaire responses or other forms of communication. We do not anticipate any risks to parents as they are simply being encouraged to participate in standard FICare activities that already occur in the participating centers. There is an emphasis that this should occur at the parents’ pace.

### Dissemination

The study findings will be presented at neonatal and FICare conferences and published in medical and nursing journals. The study findings will also be shared with relevant charities (eg, Bliss), and the study participants if they wish. It will form the basis of a PhD thesis. The PAG will be involved in the dissemination of the study findings.

### Ethical Considerations

Participants will be able to withdraw from the study at any point. Anonymized data gathered until this point will be retained for data analysis. Following each interview or focus group, parents will be given 2 weeks to withdraw their data from the study. The study was approved by Leicester, South Research Ethics Committee (22/EM/0140)in August 2022, and written consent will be obtained from participants (see participant information leaflets and consent forms in Multimedia Appendices 1 and 2).

## Results

The study is currently ongoing. As of October 2023, 22 families, comprising 22 fathers and 17 mothers had been recruited. The SR studies have been registered with the PROSPERO database (324275 and 306760). The projected end date for data collection is around July 2024, data analysis will occur in November 2024, and publication of the study findings will occur in early 2025.

## Discussion

This study will test the hypothesis that if fathers are facilitated to engage more with their baby on the NU and are better supported during the neonatal stay, both parents will experience fewer MH symptoms during their baby’s hospitalization and up to 6 weeks postdischarge. Improving parents’ MH is shown to allow greater parent-child bonding [32] and more sensitive responsiveness of mothers to their infants’ cues [33]. Mothers engaged in the FICare model have greater confidence in caregiving at the point of discharge and seek less medical support after discharge [32]. It is anticipated that these results will translate to fathers or partners.

Unlike many other studies [12,33], this study includes parents of a wide gestational age range of premature infants, including extremely premature infants and those who are invasively ventilated and at the highest risk of adverse outcomes. This is vital as research has found that fathers find the fragile appearance of their premature infants causes significant stress [34]. A focus of the study methods will be to upskill NU staff to increase awareness and understand the importance of father engagement in FICare and their MH as well as the impact on mother and child, which is in line with the NHS Long Term Plan [17].

Limitations of the study include the need for conversational English. This is required to be able to complete the standardized questionnaires, participate in the interviews and focus groups, and to be able to engage with the phase 2 provisions. The language for the phase 2 materials will be kept as simple to ensure readability and understanding. Exclusion from recruitment based on language will be monitored. The perceived research burden may disproportionately impact parents who have older children, have a greater distance to travel, limited parental leave, or who do not have English as their first language. There may also be cultural barriers to overcome when discussing MH.

A prospective cohort study was the most practical and ethical study design. The sample size and the primary outcome of the study is based on the qualitative data. A bigger sample size will be required for the study to be based on the quantitative or questionnaire data. The quantitative or questionnaire data collected in this study is to support the qualitative data and to test the feasibility of collecting such data longitudinally.

The PSS:NICU questionnaire has been translated into many languages across the world [35,36], and is a well-used tool in neonatal research. It has been validated in mothers and fathers of NICU babies in the United Kingdom [37]. There are limited validated tools to assess paternal and maternal anxiety and depression in the postpartum or NU period. Therefore, simple screening tools that are validated in primary care and the general population for patients with a variety of backgrounds and stages of life were chosen [27,38,39].

## Appendix

Multimedia Appendix 1 Participant information leaflet.

Multimedia Appendix 2 Consent form.

## Abbreviations

FICare: family integrated care
GAD-7: Generalized Anxiety Disorder-7
MH: mental health
NHS: National Health Service
NU: neonatal unit
PAG: parent advisory group
PHQ-9: Patient Health Questionnaire-9
PI: principal investigator
PSS: NICU: Parental Stressor Scale: Neonatal Intensive Care Unit
SR: systematic review

## Data Availability

The deidentified data sets generated or analyzed during this study are available from the corresponding author on reasonable request.
