# Correction: Investigating Father or Partner Involvement in Family Integrated Care in Neonatal Units: Protocol for a Prospective, Multicenter, Multiphase Study

**DOI:** 10.2196/58848

**Published:** 2024-04-03

**Authors:** Rupa Rubinstein, Katie Gallagher, John Ho, Julian Bose, Minesh Khashu, Narendra Aladangady

**Affiliations:** 1 Neonatal Unit Homerton Healthcare NHS Foundation Trust London United Kingdom; 2 Blizard Institute Queen Mary University London London United Kingdom; 3 Institute of Women's Health University College London London United Kingdom; 4 Neonatal Unit Whipps Cross University Hospital Barts Health London United Kingdom; 5 Inspire Cornwall Community Interest Company's DadPad The Health and Wellbring Innovation Centre Truro United Kingdom; 6 Neonatal Unit University Hospitals Dorset NHS Foundation Trust Dorset United Kingdom

In “Investigating Father or Partner Involvement in Family Integrated Care in Neonatal Units With TARGET (Fathers and Partners in Family Integrated Care): Protocol for a Prospective, Multicenter, Multiphase Study” (JMIR Res Protoc 2024;13:e53160), an incorrect title was published.

The title

Investigating Father or Partner Involvement in Family Integrated Care in Neonatal Units With TARGET (Fathers and Partners in Family Integrated Care): Protocol for a Prospective, Multicenter, Multiphase Study

has been revised to:

Investigating Father or Partner Involvement in Family Integrated Care in Neonatal Units: Protocol for a Prospective, Multicenter, Multiphase Study

The reason for this change is that “TARGET” is not defined nor used anywhere in the paper.

The correction will appear in the online version of the paper on the JMIR Publications website on April 3, 2024, together with the publication of this correction notice. Because this was made after submission to PubMed, PubMed Central, and other full-text repositories, the corrected article has also been resubmitted to those repositories.

