# Supplementary material for: Investigating Father or Partner Involvement in Family Integrated Care in Neonatal Units: Protocol for a Prospective, Multicenter, Multiphase Study
Source: JMIR Res Protoc. 2024 Mar 25;13:e53160. doi: 10.2196/53160 (PMC10990416; doi:10.2196/53160)
Supplement: Multimedia Appendix 1 [file resprot_v13i1e53160_app1.docx]

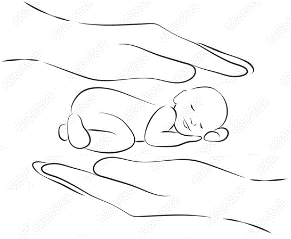


NHS TRUST LOGO

**TARGET Study- faThers And paRtners in family inteGrated carE study: Phase 2**

**Parent Information Sheet**

We would like to invite you to take part in our research study. Before you decide, it is important that you understand why the research is being done and what this would involve for you.

Please take your time to read this information leaflet and discuss with others if you wish. We can answer any questions you may have or provide further information if needed.

**What is the purpose of the study?**

Family Integrated Care (FIC) is a principle whereby parents/guardians are collaborators in their baby’s care in the neonatal unit, rather than bystanders. Mothers are more often integrated than *fathers despite both parents suffering from stress and anxiety during their baby’s admission. Many large studies focus primarily on mothers rather than fathers.

In this second phase of the study, the information gathered from phase 1, a literature review and in consultation with our Parents Advisory Group, we have developed simple intervention strategies (e.g. bite-size classes, father peer support & feeding baby) to enhance father’s involvement in FIC. Phase 2 study aims to investigate whether applying this intervention strategy will help to improve overall well-being of parents and there by improve parent-infant bonding. This will be measured through questionnaires, interviews and focus groups.

***Please note that although the study paperwork refers to ‘fathers’, all second parents of any gender or relationship status are included under this term.**

**Who has organised, sponsored and reviewed this research?**

This study has been organised by a multi-disciplinary research team and sponsored by the Homerton Healthcare NHS Foundation Trust. The study protocol has been reviewed and approved by the Leicester, South Research Ethics Committee.

**Why have I been invited?**

The study is looking at the neonatal unit journey experiences of parents with a preterm baby born more than 7 weeks early. You have been asked take part because you have a preterm baby born at less than 33 weeks gestation (more than 7 weeks early) admitted to neonatal unit or are expecting one.

**Do I have to take part?**

No, you do not have to take part in the study. Participation in the study is entirely voluntary. If you agree to take part in the study now, you can change your mind later without needing to give a reason. Declining to participate or withdrawing from the study will not impact the medical care your baby or support you receive. Participation of both parents would be ideal however the father’s participation is an essential part of the study.

If you decide to take part, we will need to your consent within 7 days of your baby’s birth.

**What will happen if I do take part?**

Participation will be required from the first week of your baby’s admission until 6 weeks post-discharge. There will also be a final baby development questionnaire to complete when your baby has their 2-year follow up appointment in the hospital.

Both parents will be asked to complete 3 short questionnaires that examine mental health (anxiety, depression and a specific questionnaire for parental stress in NICU) every 3 weeks. These tick-box questionnaires should take no more than 15 minutes to complete in total, and these are standard questionnaires used in the NHS.

Both parents will also be asked to complete the same questionnaires at 6 weeks after discharge of their baby from the neonatal unit.

We will ask most fathers to participate in 45-minute admission and pre-discharge interviews. We will also run focus groups for mothers.

| **Intervention** | **When** | **How Long** | **Who?** |
| --- | --- | --- | --- |
| GAD7:  Generalised Anxiety Disorder 7-item Questionnaire | 1^st^ week of admission and every 3 weeks thereafter until discharge | 2-3 minutes | Both parents |
| PHQ9:  Personal Health 9-item Questionnaire | 1^st^ week of admission and every 3 weeks thereafter until discharge | 2-3 minutes | Both parents |
| PSS:NICU  Parental Stressor Scale: Neonatal Intensive Care Unit Questionnaire | 1^st^ week of admission and every 3 weeks thereafter until discharge | 10 minutes | Both parents |
| Interview | 1^st^ week of admission and last week of admission | Maximum 1 hour | Fathers |
| Focus Group | During hospital admission | Maximum 1 hour | Mothers |
| GAD7, PHQ9 and PSS:NICU | 6 weeks post-discharge | 15 minutes | Both parents |
| Telephone Interview | 6 weeks post discharge | 20 minutes | Both parents |
| PARCA-R:  Parent Report of Children’s Abilities- Revised Questionnaire | To be completed at the 2 year hospital development check. | 20 minutes | A parent |

The questionnaires can be given to you in the neonatal unit or emailed to you, whichever you prefer.

Your baby’s clinical outcomes at discharge and at 2 years of corrected age (2 years old based on their expected due date) will be gathered from routinely collected hospital clinical records.

In addition to the above monitoring, this second phase of the study involves providing support for the parents, and father in particular, to participate in their baby’s care. This will primarily involve working with the nurses looking after your baby to learn how to take care of your baby even though they are small and in an incubator. This will be done at **your** pace. For example, you will learn how to change nappies in the incubator, provide mouth cares, test the nasogastric tube (tube running from nose to tummy) and give milk feeds and how to read the monitors. You can monitor your learning and formally sign off your skills in your own workbook.

There will be an app to allow you to learn more about the new world of neonatal intensive care, the equipment and medical terminology.

There will be bite-size classes run in the unit at a variety of time from different members of the neonatal team eg dieticians, breastfeeding support workers and discharge coordinators to help you provide the right support for your baby.

Father peer support sessions will be run on one evening per week to allow them to meet other fathers and discuss their own experiences in a safe, confidential and supportive environment.

The focus groups will be small group discussions for mothers led by a researcher. It will cover various aspects of having a baby in NICU, including sources of support and stress for both parents. These conversations must be kept confidential and must not be repeated outside of the room.

**What should you consider?**

You should consider the time commitments requested of you- approximately 15 minutes every 3 weeks to complete the questionnaires, and the post-discharge telephone and paper questionnaires. Fathers will be asked to commit an additional maximum 2 hours (in total) for admission and discharge interviews.

Mothers would be asked to commit to attending a focus group (maximally 1 hour) during the NICU admission.

**Are there any risks or benefits?**

**Risks:** The questionnaires and interviews may be emotionally challenging. Participation in the study may therefore cause the parents to be more aware of their psychological distress during this stressful period but support is available for all families. We can sign post you to local and national resources to support you if you wish. It may feel laborious to have many sessions available to attend on the unit such as education and psychology/peer support which may add to parental guilt if you are unable to attend.

**Benefits:** Participation in this study will help to: 1) improve parents’ awareness of how neonatal unit functions and basic knowledge of common clinical problems of babies, 2) Encourage and prepare both parents to actively participate in delivering quality family integrated care, 3) The interventions may improve the overall well-being (feeling confident & less stress) of both parents, and better parent-infant bonding and 4) The FIC interventions may improve breast feeding rate, short and long-term outcomes of babies.

**Will my study participation be kept confidential?**

We will use information from you, and your baby’s medical records for this research project.

This information will include:

- your name and contact details (telephone number and postal and/or email addresses as is your preference)
- your anonymised answers from your questionnaires,
- your baby’s NHS number to get their medical information

People will use this information to do the research or to check your records to make sure that the research is being done properly. People who do not need to know who you are will not be able to see your name or contact details. Your data will have a code number instead. We will keep all information about you safe and secure.

You can stop being part of the study at any time, without giving a reason, but we will keep information about you that we already have. We need to manage your records in specific ways for the research to be reliable. This means that we won’t be able to let you see or change the data we hold about you.

You can find out more about how we use your information at [www.hra.nhs.uk/information-about-patients/](https://www.hra.nhs.uk/information-about-patients/), by asking one of the research team members, sending an email to [n.aladangady@nhs.net](mailto:n.aladangady@nhs.net) or [rupa.rubinstein@nhs.net](mailto:rupa.rubinstein@nhs.net) , or by ringing us on 020 8510 7361/7364. You can also contact the Data Protection Officer at email address - [huh-tr.ig@nhs.net](mailto:huh-tr.ig@nhs.net) or telephone number 02076834102.

All questionnaire data shall be put into an anonymised table, and the original paper or electronic copies destroyed as per NHS policy/guidelines. Audio recordings of the interviews and focus groups shall be transcribed, and any identifiable data removed from transcriptions after which the recording will be deleted.

At the end of the study, we will save some of the data in case we need to check it and for future research. We will make sure no-one can work out who you are from the reports we write. Anonymised direct quotes may be used in research publications.

Please note that confidentiality may be broken in the case of criminal disclosures or to prevent harm to yourself or another person.

**What happens if I don’t want to participate in the study anymore?**

Participation in the study is voluntary and you are free to change your mind at any point. Any information already collected will be used for research purposes. Your baby’s medical care or support offered to you shall not be impacted in any way. After your interview/focus group you will have 2 weeks to withdraw your consent before the recording is fully anonymised and integrated into the study data for analysis.

**What happens at the end of the study?**

The study findings shall be presented at neonatal and Family Integrated Care conferences. We are aiming to publish the research findings in medical journals. The study findings will be shared with other neonatal units across the UK through Neonatal Networks and relevant charity bodies.

This research is part of an educational project and shall also contribute to the fulfilment of a PhD thesis.

Study participants shall not be identifiable through any future presentations or publications of research findings.

**Have parents and members of the public been involved?**

The study team includes a member of the public and veteran NICU parents. Our lay parent panel have reviewed the study design and all the study papers you will see, including this sheet.

**What if there is a problem?**

If you wish to complain or have concerns about any aspect of the study or the way you have been treated during the study, you should contact the investigators (see below details). Alternatively, you can contact the hospital Patient Advice and Liaison Service (PALS). They can be contacted at Homerton at 02085105522 or can be found at the main entrance.

*Professor Narendra Aladangady*

*Consultant Neonatologist and Chief Investigator*

*Homerton University Hospital*

*Tel – 0208 510 7360*

*Dr Rupa Rubinstein*

*Clinical Research Fellow and Principal Investigator*

*Homerton University Hospital*

*Tel – 0208 510 7361/7364*

**Thank you for reading this information leaflet**

**The doctor or nurse who gave you this leaflet will be pleased to discuss the study in more detail and provide further information if this would be helpful. Alternatively, the contact details of the study’s Chief and Principal Investigators are provided on this page.**
