# Supplementary material for: Investigating Father or Partner Involvement in Family Integrated Care in Neonatal Units: Protocol for a Prospective, Multicenter, Multiphase Study
Source: JMIR Res Protoc. 2024 Mar 25;13:e53160. doi: 10.2196/53160 (PMC10990416; doi:10.2196/53160)
Supplement: Multimedia Appendix 2 [file resprot_v13i1e53160_app2.docx]

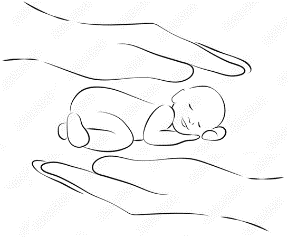


NHS TRUST LOGO

**TARGET Study- faThers And paRtners in family inteGrated carE study: Phase 2**

**Parent Consent Form**

Please check the boxes if you agree. Initials

| I confirm that I have read and understand the ‘Fathers and Partners in Family Integrated Care’ study information sheet (v5.0 dated 31/03/2023) and that I have been given the opportunity to ask any questions and have received satisfactory answers. |  |
| --- | --- |
| I understand that my participation in the study is voluntary and may be withdrawn at any time for any reason without the medical or nursing care of my baby being impacted, or legal rights being affected. I understand that I can withdraw my interview or focus group data up to 2 weeks after the event. |  |
| I understand that sections of the baby’s medical notes may be looked at by the research team, authorised representatives from the sponsor (as part of their auditing responsibilities) or from the regulatory authorities where it is relevant to the study outcomes. I give permission to these individuals to have access to these records. |  |
| I understand that the medical care of my baby shall not be altered in any way by participation in this study. |  |
| I understand that the interviews and focus groups will be audio-taped and held on a secure server. Once transcribed and verified, the original recording will be immediately destroyed. I have 2 weeks to withdraw my consent for the recording to be used in the study. |  |
| I agree to participate in the study. |  |

______________________ ______________________ ______________________

Name of Parent Signature Date

______________________ ______________________ ______________________

Name of person taking consent Signature Date

(if not PI)

______________________ ______________________ ______________________

Principal Investigator Signature Date
